# Supplementary material for: The role of accelerometer-derived sleep traits on glycated haemoglobin and glucose levels: a Mendelian randomization study
Source: Sci Rep. 2024 Jun 28;14:14962. doi: 10.1038/s41598-024-58007-9 (PMC11213880; doi:10.1038/s41598-024-58007-9)
Supplement: Supplementary file 2 — Supplementary Figures. [file 41598_2024_58007_MOESM2_ESM.doc]

# **Title**

**The role of accelerometer-derived sleep traits on glycated haemoglobin and glucose levels: a Mendelian randomization study**

**Supplementary figures**

**Supplementary Figure S1 The one-sample Mendelian randomization estimates of self-reported (SR) sleep traits with: a) HbA1c and b) non-fasting glucose in European UKB participants with accelerometer-derived sleep data, and in all available White British UKB participants.**


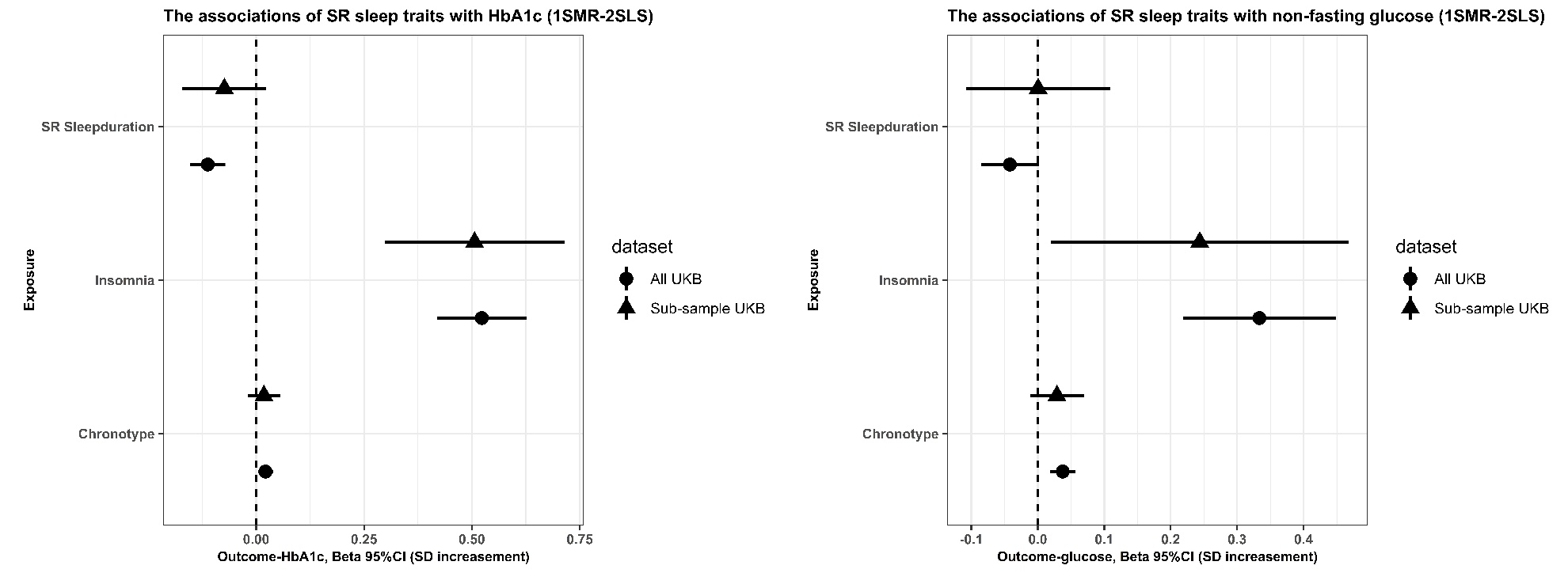


1SMR-2SLS: one-sample MR with two-stage least square method.

SR sleep duration: per hour increase

Insomnia: usually experiencing insomnia symptoms vs sometimes, rarely/never

Chronotype: per category increase to evening preference

1SD HbA1c in the whole UKB is 0.15 log mmol/mol; 1SD HbA1c in the sub-sample of UKB is 0.14 log mmol/mol

1SD glucose in the whole UKB is 0.17 log mmol/l; 1SD glucose in the sub-sample of UKB is 0.15 log mmol/l

Glucose in the UK Biobank was non-fasting.

**Supplementary Figure S2 The Pearson correlations across accelerometer-derived and self-reported sleep traits in the European UKB participants with accelerometer-derived sleep data**

**
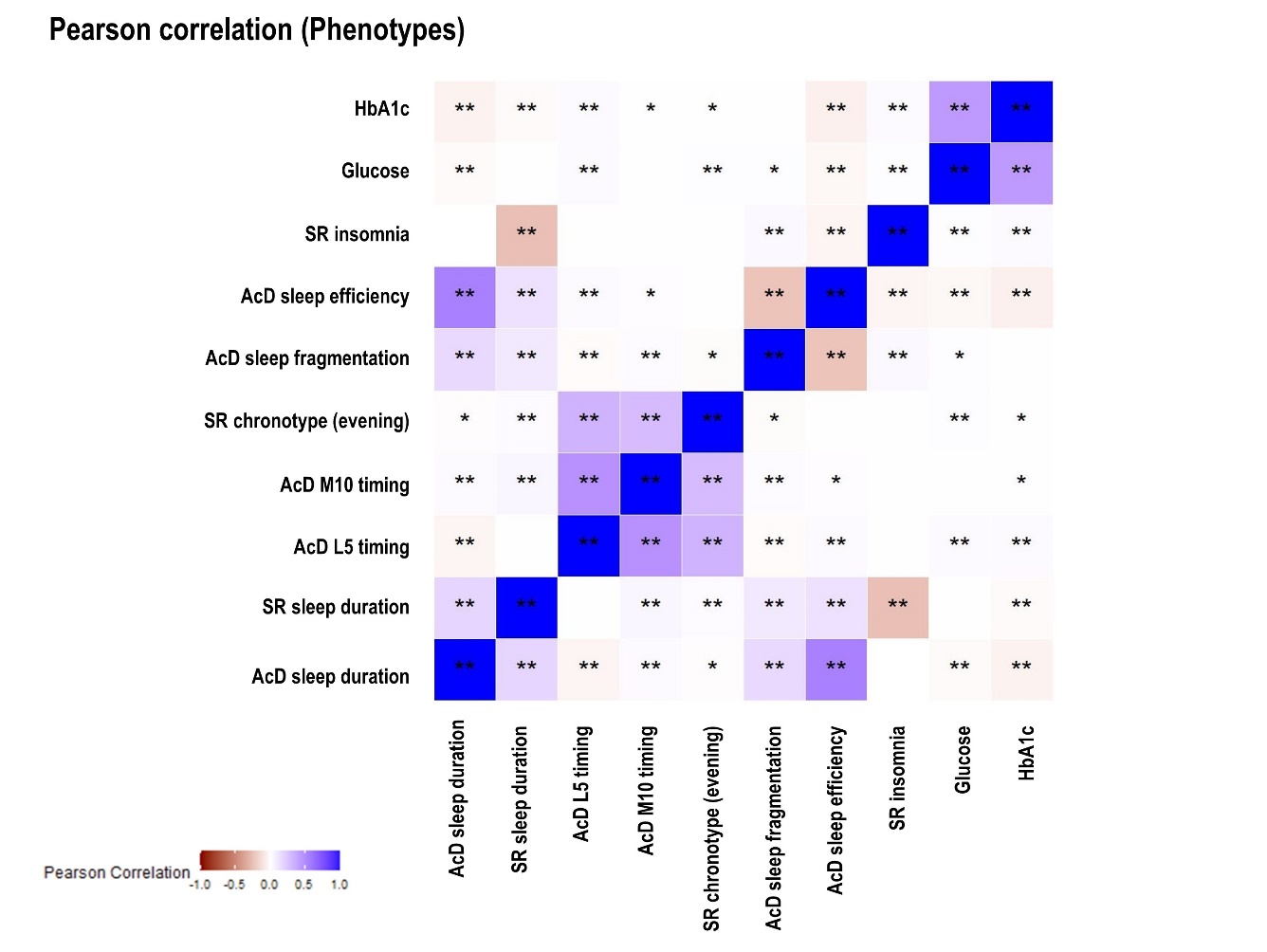
**

Legends:

* p-value < 0.05

** p-value < 0.001
